# Seagrass morphometrics at species level in Moreton Bay, Australia from 2012 to 2013

**DOI:** 10.1038/sdata.2017.60

**Published:** 2017-05-09

**Authors:** Jimena Samper-Villarreal, Chris Roelfsema, Eva M. Kovacs, Novi S. Adi, Mitchell Lyons, Peter J. Mumby, Catherine E. Lovelock, Megan I. Saunders, Stuart R. Phinn

**Affiliations:** 1 Marine Spatial Ecology Lab and ARC Centre of Excellence for Coral Reef Studies, University of Queensland, St Lucia, Queensland 4072, Australia; 2 CSIRO Ocean and Atmosphere Flagship, Ecosciences Precinct, Brisbane, Queensland 4001, Australia; 3 Centro de Investigación en Ciencias del Mar y Limnología (CIMAR) & Escuela de Biología, Universidad de Costa Rica, San Pedro, 11501-2060 San José, Costa Rica; 4 Remote Sensing Research Centre, School of Earth and Environmental Sciences, University of Queensland, St Lucia, Queensland 4072, Australia; 5 Centre for Marine Research, Ministry of Marine Affairs and Fisheries, Jl. Pasir Putih 2, Ancol Timur, Jakarta 14430, Indonesia; 6 Centre for Ecosystem Science, University of New South Wales, Sydney, New South Wales 2052, Australia; 7 School of Biological Sciences, University of Queensland, St Lucia, Queensland 4072, Australia; 8 Global Change Institute, University of Queensland, St Lucia, Queensland 4072, Australia

**Keywords:** Natural variation in plants, Natural variation in plants, Natural variation in plants, Natural variation in plants, Natural variation in plants

## Abstract

Seagrass above, below and total biomass, density and leaf area, length and width were quantified at a species level for 122 sites over three sampling periods in Moreton Bay, Australia. Core samples were collected in two regions: (1) a high water quality region with varying species assemblages and canopy complexity (98 sites); and (2) along a turbidity gradient in the bay (24 sites within four locations). Core samples were collected using a 15 cm diameter×20 cm long corer. Seagrass dry biomass per component was quantified per species present in each sample. A total of 220 biomass and density data records are included, 130 from the high water quality region and 90 from the turbidity gradient. These data provide a detailed assessment of biomass, density and leaf metrics per species sampled from Moreton Bay over 2012–2013. In future, these can be used as a baseline to assess seasonal and spatial variation within the bay, within the region and among regions.

## Background & Summary

This data set was collected to quantify seagrass biomass, density and leaf metrics along a turbidity gradient and in an area of high water quality in Moreton Bay, during a total of three sampling periods over two years. The data were used to analyse spatial and seasonal variation in seagrass morphometrics, determine seagrass canopy complexity, as an explanatory variable for seagrass sediment carbon content, and determine above and below ground seagrass carbon pools^1^. Seagrass biomass data from the high water quality area, the Eastern Banks, was integrated with seagrass cover and species map products generated from satellite imagery and coincident benthic field data. Collectively, high resolution maps representing seagrass biophysical parameters (species, cover, and biomass as a function of species and cover) were generated^2–4^.

## Methods

### Sampling design

**Figure 1:**
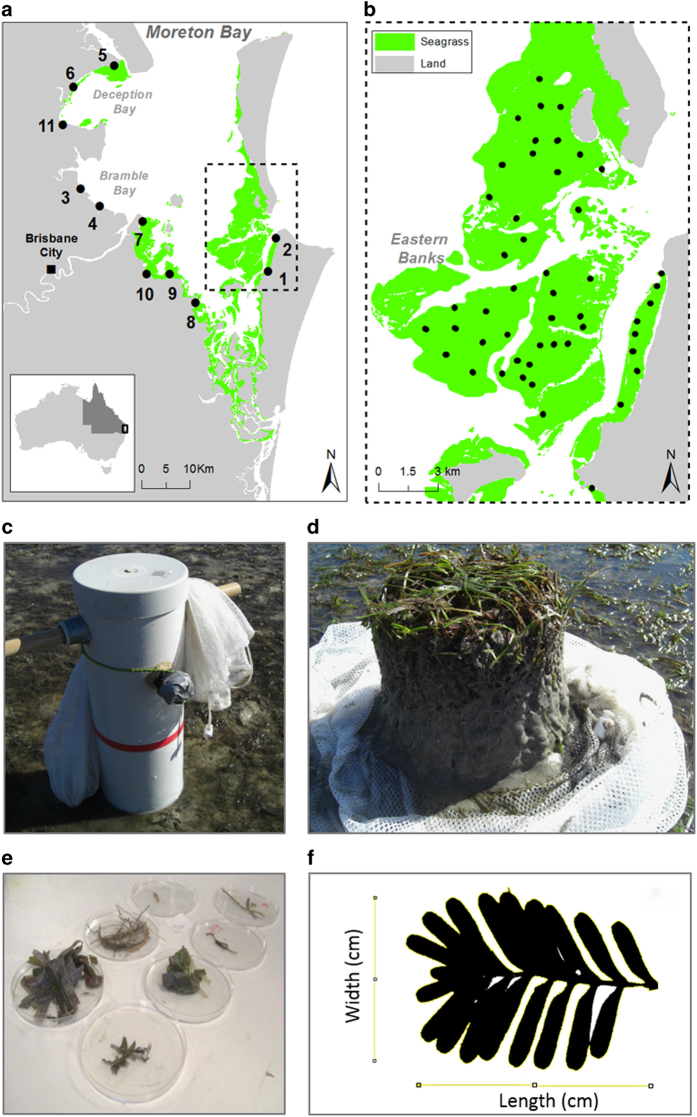
Location of study sites, field photos of core sampling, separation into biomass components and measurement of leaf metrics from seagrass meadows in Moreton Bay, SE Queensland, Australia. (**a**) Field sampling locations (black dots) across a *Turbidity Gradient*, with sampling on the western side with low water quality and on the northern and eastern sides of Moreton Bay with higher water quality; (**b**) sampling sites (black dots) within the Eastern Banks, an area of varying *Canopy Complexity* and high water quality; (**c**) coring device used to collect core samples and mesh for rinsing off sediment at each sampling site; (**d**) core sample collected prior to rinsing using the mesh; (**e**) separate biomass components from a core sample (g DW m^−2^); and (**f**) leaf image used to estimate leaf length, width and area at species level using ImageJ. Numbers refer to sample sites at: (1) Myora; (2) Amity; (3) Sandgate; (4) Nudgee; (5) North Deception Bay; (6) Beachmere; (7) Port of Brisbane; (8) Cleveland; (9) Wellington Point; (10) Lota; & (11) South Deception Bay. Note: **a**,**b**) shaded green area represent seagrass distribution in June 2011 (Data Citation 1).

This data set was developed by collecting seagrass core samples in two sampling regions in Moreton Bay, South East Queensland, Australia. Samples were collected from the Eastern Banks, where water quality is relatively high and spatially consistent; and in an area where water quality varies across a spatial gradient (Fig. 1). Core samples were collected during three sampling periods over 2012–2013. In the austral winter of 2012 individual core samples were collected from 98 sites in the Eastern Banks (Fig. 1). In winter 2012, six individual core samples were also collected at random from a 50×50 m plot at each of eleven locations in the water quality gradient (Fig. 1). In the austral summer of 2013, 32 core samples were collected from a subset of the original sites in the Eastern Banks. In the austral summer and again in the winter of 2013, 48 core samples were collected from a subset of eight locations along the water quality gradient (Fig. 1). At each site, one seagrass core sample was collected for the assessment of species composition, biomass, and seagrass morphometrics. GPS coordinates were obtained for each collection site and are included in the dataset.

The Eastern Banks region has lush seagrass meadows and the highest diversity of species relative to the greater Moreton Bay. Depth at high tide at the Eastern Banks sites ranged from 0.8 to 3.7 m. Sampling sites were accessed by snorkelling when the water column was greater than 1 m depth. These sites included seagrass communities with varying assemblages and structural complexities and were selected to represent heterogeneity of seagrass composition based on remotely sensed data^4^ (Fig. 1). Locations along the water quality gradient were selected to be representative of the spatial variability in water quality within the bay^1,5^. The sampling scheme for the water quality gradient included four unvegetated sites within the area of modelled potential seagrass habitat based on benthic light availability and wave height^1,6^, which have been reported to have seagrasses in the past^5^. At the time of survey, these sites did not have seagrass present and are therefore not included in this data set (Sandgate, Nudgee, Beachmere, and South Deception Bay). More information on these sites can be found in the publications linked to this dataset^1^. Seagrass meadows along the water quality gradient were dominated by two species: *Zostera muelleri* and *Halophila ovalis*.

### Seagrass coring, species composition, biomass, and morphometrics

At each site one core sample was collected for the quantification of seagrass species composition, biomass, and morphometrics. Core dimensions were 15 cm diameter×20 cm length. The corer used in the turbidity gradient was a PVC hollow tube with a sealed PVC lid, wooden handles and a small lateral opening and corresponding cap to create a vacuum seal when collecting the sample (Fig. 1). Corer used in the high water quality sampling was the same, except without the top and opening with cap. The two corers were selected as the most effective coring devices given the type of sediment at each site, the depth the samples were collected at, and logistics in the field. Each core sample was removed intact from the sediment, collecting 20 cm depth of sediments consistently among sites. Core samples were then gently rinsed free of sediment in the field using a 1 mm mesh bag in sea water to retain seagrass material. Core samples were kept on ice in the field, then stored frozen (−20 °C) until further processing. Subsequently, core samples were thawed in cold water and the species present in each sample noted. The number of shoots per species in each core was quantified to estimate shoot density per species (shoots m^−2^). Biomass material from each core was separated per species into above ground (leaves and leaf stems) and below ground (roots, rhizomes, and leaf sheaths) material. Prior to drying, leaves were submerged in 10 % hydrochloric acid (HCl) and rinsed with fresh water to remove calcareous epiphytes. Foliar epiphytes were gently scraped off using laboratory forceps. Biomass material of macroalgae and mangrove vegetation found was also separated. Detrital material was separated from the sample using a 0.5 mm mesh after all other components were isolated. Each component was then dried at 60 °C and final biomass (g dry weight (DW) m^−2^) calculated.

## Data Records

The seagrass morphometric data at species level from both sampling regions and the three sampling seasons has been compiled into one dataset. The data has been archived in tab-delimited and html format at Pangaea.de [Data Citation 2]. The file contains both collection and seagrass characteristics. Collection information details the date of collection, specific location within Moreton Bay, South East Queensland including GPS coordinates, and the specific name assigned to each site (Table 1). Seagrass parameters included are the seagrass shoot density, mean leaf length, width and area per species, and above, below ground and total biomass (Table 1).

## Usage Notes

The records provided in this dataset refer to seagrass morphometric values which can provide a basis for seagrass morphometric comparison with other sites within Moreton Bay or those in other regions. Future comparison can analyse seagrass species composition, total, above and below ground biomass per species and in total, as well as shoot density per species and in total, and leaf area, length and width, per species and in total. This dataset includes values from three separate sampling times in 2012 and 2013, and can be compared in future with other morphometric studies at other temporal scales. This dataset has already been used for comparison with carbon storage in these meadows^1^, and linked with *in situ* and satellite imagery for much finer spatial and temporal scales of study^3,4^. Current modelling using these data is underway but ample modelling could be developed using this dataset, in particular in conjunction with other datasets from other regions. The records here include morphometrics at multiple level of detail for usage, from the total biomass, density and leaf metrics, to those down to each individual species allowing the data to be useful for multiple types of analyses in future.

## Additional Information

**How to cite this article:** Samper-Villarreal, J. *et al.* Seagrass morphometrics at species level in Moreton Bay, Australia from 2012 to 2013. *Sci. Data* 4:170060 doi: 10.1038/sdata.2017.60 (2017).

**Publisher’s note:** Springer Nature remains neutral with regard to jurisdictional claims in published maps and institutional affiliations.

## Supplementary Material



## Figures and Tables

**Table 1:** Structure and nomenclature of the categories denoted for each seagrass morphometric data record (Data Citation 2).

**Record Structure**	**Detailed categories denoted for each record**
Site location for each sample	Date collectedRegion and subregionSite nameGPS coordinated (Longitude & Latitude)
Biomass (BMS) (g DW m^−2^)	Above ground biomass (AG) per species and total for the sampleBelow ground biomass (BG) per species and total for the sampleTotal biomass for the sampleSpecies per sample: *Zostera muelleri* (ZM), *Halophila ovalis* (HO), *Halophila spinulosa* (Hs) *Cymodocea serrulata* (Cs), *Halodule uninervis* (Hu), *Syringodium isoetifolium* (Si) Other categories per sample: detritus, macroalgae, mangrove
Density (shoots m^−2^)	Species per sample: *Zostera muelleri* (ZM), *Halophila ovalis* (HO), *Halophila spinulosa* (Hs) *Cymodocea serrulata* (Cs), *Halodule uninervis* (Hu), *Syringodium isoetifolium* (Si)Total density per sample
Leaf metrics	Average leaf area (cm^−2^)Average leaf length (cm)Average leaf width (cm)Species per sample: *Zostera muelleri* (ZM), *Halophila ovalis* (HO), *Halophila spinulosa* (Hs) *Cymodocea serrulata* (Cs), *Halodule uninervis* (Hu), *Syringodium isoetifolium* (Si)

## References

[d1] PANGAEA Roelfsema C. M. 2014 https://doi.org/10.1594/PANGAEA.833767

[d2] PANGAEA Samper-Villarreal J. 2016 https://doi.org/10.1594/PANGAEA.864316

